# A machine learning algorithm for automatic tumour board recommendations in prostate cancer patients

**DOI:** 10.1002/bco2.70066

**Published:** 2025-08-18

**Authors:** Marcus Sondermann, Hannah Glaser, Anke Rentsch, Katharina Boehm, Roman Herout, Tobias Hölscher, Fabian Lohaus, Fabian Funer, Matthias Miederer, Christian Thomas, Sherif Mehralivand

**Affiliations:** ^1^ Department of Urology University Hospital Carl Gustav Carus, TU Dresden Dresden Germany; ^2^ Medical Faculty Philipps University Marburg Marburg Germany; ^3^ National Center for Tumor Diseases (NCT), University Hospital Carl Gustav Carus, TU Dresden Dresden Germany; ^4^ Department of Radiotherapy and Radiation Oncology University Hospital Carl Gustav Carus, TU Dresden Dresden Germany; ^5^ Department of Nuclear medicine University Hospital Carl Gustav Carus, TU Dresden Dresden Germany

**Keywords:** artificial intelligence, decision support, machine learning, prostate cancer, tumour board

## Abstract

**Background and objective:**

Multidisciplinary tumour boards (MTBs) play a critical role in prostate cancer management, but their time‐intensive nature limits accessibility. This study evaluates machine learning (ML) algorithms for automating MTB recommendations in prostate cancer patients, focusing on multi‐label classification for diagnostic and therapeutic decisions.

**Methods:**

A retrospective dataset of 1929 MTB recommendations from 2020 to 2024 was used for model development and validation at a single academic centre. Three ML algorithms—Decision Tree, Random Forest and K‐Nearest Neighbours (KNN)—were trained to predict recommendations for PSMA‐PET, conventional imaging, active surveillance and local therapy (radical prostatectomy or radiotherapy). Model performance was assessed using accuracy, precision, recall and F1‐score.

**Key findings and limitations:**

The Random Forest model achieved the highest overall accuracy (66.3%, 95% CI 61.7–71%) and showed stable performance across most outcome categories. Predictions for local therapy were highly accurate (F1‐score: 0.99), but model performance was lower for less frequent recommendations such as PSMA‐PET and active surveillance, reflecting class imbalance and recent guideline changes. Limitations include moderate overall accuracy, retrospective single‐centre design and the need for extensive manual data preprocessing. In addition, a high proportion of patients were eligible for multiple treatment options, which may limit the discriminatory value of certain outcomes.

**Conclusions and clinical implications:**

This study demonstrates the potential of ML to replicate MTB decision patterns in prostate cancer with reasonable accuracy. However, the current model requires further optimization before it can be considered for clinical application. It should be regarded as a proof‐of‐concept that highlights both the opportunities and the challenges of algorithm‐based decision support in oncology. Future work should focus on improving model performance through multi‐institutional data, prospective validation and continuous adaptation to evolving clinical guidelines.

## 1 INTRODUCTION

Prostate cancer is the most common malignancy among men and the second leading cause of cancer‐related mortality worldwide.^1^ While early detection through prostate‐specific antigen (PSA) screening has reduced mortality rates,^2^ it has also led to challenges such as overdiagnosis and overtreatment. Prostate cancer exhibits a highly heterogeneous disease course, ranging from indolent tumours requiring active surveillance to aggressive malignancies demanding immediate intervention. Consequently, accurate risk stratification and personalized treatment recommendations are critical to optimizing oncological outcomes while minimizing unnecessary treatment‐related morbidity.

Multidisciplinary tumour boards (MTBs) have become a cornerstone in modern oncological decision‐making, enabling collaborative discussions among specialists in radiology, nuclear medicine, pathology, surgery and oncology. Their approach enhances adherence to guidelines and has been associated with improved clinical outcomes and patient satisfaction.^3^, ^4^ However, MTBs are limited to high‐volume centres due to the substantial administrative and logistical burden they impose. The increasing complexity of patient data further exacerbates these challenges, underscoring the need for automated decision‐support systems.^5^

Artificial intelligence (AI) demonstrated the potential to streamline oncological workflows by leveraging machine learning (ML) algorithms for automated decision‐making. Classical ML models displayed strong performance in structured clinical datasets while requiring fewer computational resources compared to deep learning (DL) approaches. Given the need for an efficient and interpretable AI solution, we developed a ML‐based algorithm for the automatic generation of MTB recommendations in newly diagnosed prostate cancer patients.

This study aimed to develop, train and validate an ML model using real‐world tumour board data from a single institution in a retropective dataset. We employed a multi‐label classification approach to predict key clinical recommendations, including imaging strategies (PSMA‐PET, conventional staging) and treatment options (radical prostatectomy, radiotherapy and active surveillance). These represent different clinical decision points – diagnostic recommendations versus treatment decisions. After assessing multiple classification algorithms, the best‐performing model was implemented into a web‐based application for demonstration and experimental testing. By integrating AI‐driven automation into tumour board workflows, we aim to improve efficiency and facilitate broader access to high‐quality oncological decision‐making.

## 2 METHODS

### 2.1 Tumour board

Patients were diagnosed with prostate cancer through our biopsy clinic or referrals with biopsy reports. All patients presented were being considered for initial management discussion at the tumour board. They were registered for the MTB by our staff or treating physician. Certified MTB staff entered relevant data into our in‐house software, TDS (Tumour Documentation System). All tumour board reports and recommendations were extracted as Word documents and saved on a hospital network drive. As this workflow is part of standard operations, Institutional Review Board (IRB) approval was waived.

### 2.2 Dataset

The patient history and recommendations from November 2007 until July 2024 were subsequently extracted. Clinical variables, including patient age, prostate‐specific antigen (PSA) levels, prostate volume, digital rectal examination (DRE) results, International Society of Urological Pathology (ISUP) category, the number of positive biopsy cores, the overall number of biopsy cores and various comorbidities such as hypertensive disease, diabetes mellitus, chronic heart disease, hyperlipoproteinemia, additional malignancies and prior surgeries, were extracted from the text body. The computational expertise for developing, testing and deploying these AI programs was provided by our co‐authors with expertise in machine learning and data science. Data extraction process was executed through the utilization of regular expressions.^6^ The feasibility of this approach is attributable to the standardized and categorical nature of the patient history and recommendations. To ensure data integrity during the development, a manual proofreading process was conducted on all documents.

The outcome was defined as a multilabel classification problem, given the potential for multiple tumour board recommendations to be in effect simultaneously. These recommendations encompassed staging examinations, such as PSMA‐PET scans, CT scans and bone scintigraphy, as well as therapy strategies, including radical prostatectomy, radiation therapy and active surveillance. Local treatment with curative intent was handled in the model as a single outcome combining both radiation therapy and surgery, as patients qualifying for one typically qualify for both options. The models were trained on this outcome.

All variables were stored in a database as continuous (e.g., age, PSA, prostate volume, number of positive and overall biopsy cores) or categorical (e.g., DRE, Gleason score and all comorbidities) variables.

### 2.3 Data preprocessing

As decision‐tree‐based models demonstrate robust performance in the absence of standardization, this step was excluded in the data preparation phase. In the initial development stage, multiple test experiments were conducted to ascertain that standardization yielded analogous results, even for the K‐Nearest‐Neighbour (KNN) model. Consequently, continuous variables were employed directly, while categorical variables were converted to numerical variables using ordinal encoding.

### 2.4 Model training and validation

A comparison of deep learning‐based approaches and classical ML models reveals that the former do not offer advantages for tabular data. However, the latter requires significantly **fewer** computational resources. Consequently, we opted to train and assess a range of ML models that were particularly well‐suited for multilabel classification tasks. The Decision Tree Classifier (DT),^7^ Random Forest (RF)^8^, ^9^ and KNN models^10^, ^11^, ^12^, ^13^ were selected based on a OneVsRest approach.

**FIGURE 1:**
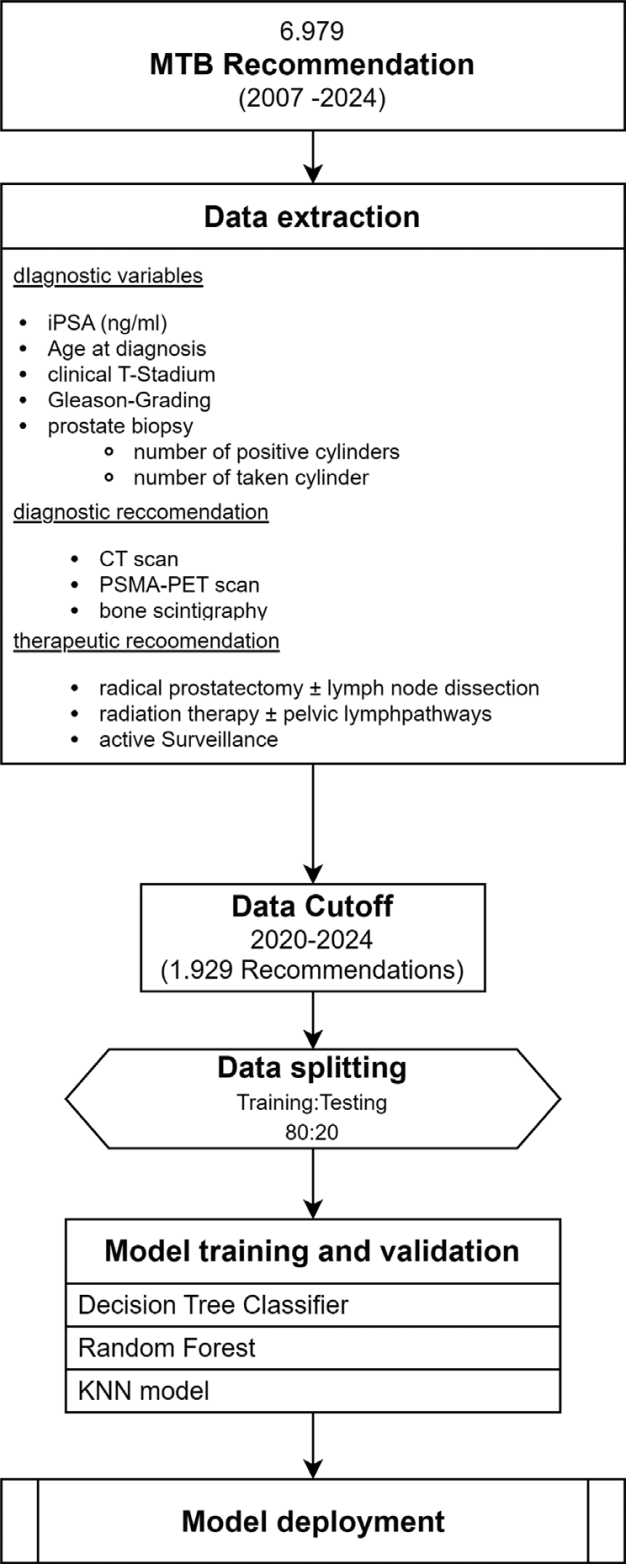
Flowchart of data extraction and model. Development pipeline overview of cohort selection, data preprocessing, outcome stratification, model training and deployment.

The dataset was partitioned into a training cohort and validation cohort, with a ratio of 80:20. Active surveillance (AS) and PSMA‐PET imaging have recently been incorporated into German prostate cancer guidelines.^14^ Preliminary experiments indicated that precision and recall for these two outcomes were low due to the limited number of recommendations in comparison to the overall size of the dataset. Consequently, we opted to incorporate exclusively tumour board sessions from 01/01/2020 onwards. Furthermore, the dataset was stratified based on the presence or absence of AS and PSMA‐PET imaging to ensure an adequate sample size for each group. The dataset procurement and split are summarized in Figure 1.

### 2.5 Model deployment

The model that demonstrated the highest accuracy was stored in pickle format for shipping and deployment. A rudimentary frontend web application was developed to load and serve the model, employing the streamlit framework.^15^ The application loads the model, and users can enter patient data. Upon selection of the prediction functionality, the prediction calculation is initiated, employing all the values entered into the system. The output vector variable is then converted to the German tumour board recommendation text using a simple heuristic text converter. The application outputs the outcome vector variable, the converted text recommendation and the prediction time.

### 2.6 Software

Python including the packages NumPy, Pandas and Scikit‐Learn were utilized for data preprocessing, training and validation.^16^, ^17^, ^18^ The complete source code is accessible on our institutional GitHub page (Link: https://github.com/ukd-uro/pretb_rf_dev). The pickle file of the model that demonstrated the highest level of performance is also available in a GitHub repository (Link: https://github.com/ukd-uro/pretb_rf_app). The web application can be accessed and used for demonstration purposes (Link: https://pretb-rf.streamlit.app).

### 2.7 Statistical analysis

Following the training phase, each model was evaluated on the validation cohort. The primary performance metrics, including overall accuracy, recall and precision, were calculated.

Overall accuracy was defined as the number of correct predictions divided by the total number of predictions made. Precision was defined per outcome class as the number of true positives divided by the sum of the number of true positives and false positives. Recall was defined per outcome class as the number of true positives divided by the sum of true positives and false negatives. The F1‐score was defined as the harmonic mean of precision and recall. To calculate 95% confidence intervals, 1000 bootstrap samples were utilized.

The statistical analysis was conducted using R version 4.3.3, with the tidyverse and gtsummary packages, and Python's scikit‐learn evaluation metrics packages.^6^, ^19^, ^20^

## 3 RESULTS

### 3.1 Data cohort

In total, 6979 tumour board recommendations were recorded between December 2007 and July 2024. A detailed overview of patient and clinical characteristics for the complete dataset is provided in Table S1. To ensure compatibility with contemporary German guideline recommendations, only cases from January 2020 onward were included in model development. This resulted in a final dataset of 1929 recommendations used for training and validation. To enhance model robustness, an 80:20 hold‐out validation approach was applied. Given the underrepresentation of “Active surveillance” and “PSMA‐PET” recommendations, stratified sampling ensured adequate representation of these categories in both training and validation cohorts. The clinical characteristics of this model cohort are summarized in Table 1. Table S2 presents the distribution of tumour board recommendations across the entire dataset (*n* = 6979) and is provided for reference.

**TABLE 1:** Clinical and demographic characteristics of the final model cohort (*n* = 1929): Distribution of baseline variables used for training and evaluation of machine learning models.

Characteristic	Overall	Training data	Validation data
	*N* = 1929^1^	*N* = 1543^1^	*N* = 386^1^
***Age***	67 (62, 71)	66 (61, 71)	67 (62, 70)
***PSA (ng/ml)***	7 (5, 11)	7 (5, 11)	7 (5, 11)
***DRE***			
*cT0*	1540 (80%)	1246 (81%)	294 (76%)
*cT1*	137 (7.1%)	108 (7.0%)	29 (7.5%)
*cT2*	38 (2.0%)	32 (2.1%)	6 (1.6%)
*cT3*	123 (6.4%)	93 (6.0%)	30 (7.8%)
*cT4*	84 (4.4%)	60 (3.9%)	24 (6.2%)
*NA*	7 (0.4%)	4 (0.3%)	3 (0.8%)
***Site of positive cores***			
*Both sided*	945 (49%)	745 (48%)	200 (52%)
*Left*	407 (21%)	331 (21%)	76 (20%)
*Right*	380 (20%)	308 (20%)	72 (19%)
*NA*	197 (10.2%)	159 (10.3%)	38 (9.8%)
***ISUP grading***			
*1*	279 (14%)	219 (14%)	60 (16%)
*2*	813 (42%)	651 (42%)	162 (42%)
*3*	350 (18%)	287 (19%)	63 (16%)
*4*	265 (14%)	214 (14%)	51 (13%)
*5*	212 (11%)	164 (11%)	48 (12%)
*NA*	10 (0.5%	8 (0.5%)	2 (0.5%)
***Number of positive cores***	4.0 (2.0, 6.5)	4.0 (2.0, 6.0)	4.0 (2.0, 7.0)
***Number of taken cores***	12.00 (12.00, 15.00)	12.00 (12.00, 16.00)	12.00 (12.00, 15.00)
***Comorbidities***			
*Hypertension*	890 (46%)	698 (45%)	192 (50%)
*Diabetes mellitus*	152 (7.9%)	120 (7.8%)	32 (8.3%)
*Cardiovascular*	72 (3.7%)	57 (3.7%)	15 (3.9%)
*Obesity*	41 (2.1%)	37 (2.4%)	4 (1.0%)
***Diagnostics***			
*PSMA scan*	291 (15%)	233 (15%)	58 (15%)
*Conventional staging*	451 (23%)	360 (23%)	91 (24%)
***Therapeutics***			
*Active surveillance*	112 (5.8%)	89 (5.8%)	23 (6.0%)
*RP or SBRT*	1919 (99%)	1535 (99%)	384 (99%)

*Note:* The superscript defines the size of the cohorts, to be compared as objective as different sizes in cohorts are relevant to understand the baselines.

### 3.2 Model training and validation

The overall predictive accuracies for the three models were as follows: DT (62.2%, 95% CI: 57.5%–66.8%), RF (66.3%, 95% CI: 61.7%–71%) and K‐Nearest Neighbours (66.6%, 95% CI: 61.7%–71.5%). The classification performances for each model across all outcome classes are presented in Table 2.

**TABLE 2:** Classification performance of machine learning models across all outcome categories: Precision, recall and F1‐score for Decision Tree, Random Forest and K‐Nearest Neighbour classifiers on the test set.

	Precision	Recall	F1‐score
***Decision Tree***			
*PSMA‐PET scan*	0.44 (0.26–0.64)	0.19 (0.09–0.3)	0.27 (0.13–0.39)
*Conventional staging*	0.67 (0.52–0.8125)	0.3 (0.2–0.39)	0.41 (0.3–0.51)
*Active surveillance*	0.63 (0.46–0.79)	0.88 (0.71–1)	0.73 (0.58–0.84)
*Prostatectomy or SBRT*	0.99 (0.98–1)	0.99 (0.98–1)	0.99 (0.98–0.99)
***Random Forest***			
*PSMA‐PET scan*	0.48 (0.31–0.63)	0.31 (0.19–0.44)	0.37 (0.24–0.5)
*Conventional staging*	0.74 (0.59–0.85)	0.36 (0.26–0.46)	0.48 (0.37–0.58)
*Active surveillance*	0.75 (0.55–0.9)	0.79 (0.6–0.95)	0.77 (0.62–0.89)
*Prostatectomy or SBRT*	0.99 (0.99–1)	1 (1–1)	0.99 (0.99–1)
***K‐Nearest Neighbours***			
*PSMA‐PET scan*	0.5 (0.29–0.69)	0.19 (0.1–0.3)	0.27 (0.15–0.4)
*Conventional staging*	0.61 (0.5–0.74)	0.38 (0.28–0.49)	0.47 (0.37–0.57)
*Active surveillance*	1 (0–1)	0.12 (0–0.29)	0.22 (0–0.44)
*Prostatectomy or SBRT*	0.99 (0.98–1)	1 (1–1)	0.99 (0.99–1)

Notably, the prediction of local therapy (radical prostatectomy or radiotherapy) yielded the highest precision, recall and F1‐scores across all models. This likely reflects the high prevalence of patients with localized prostate cancer and the fact that both treatment options are frequently guideline‐concordant for patients with localized disease. As a result, a large proportion of patients qualified for both modalities, which limits the discriminatory power of this outcome in model training. The low prevalence of PSMA‐PET and active surveillance recommendations, on the other hand, contributed to reduced performance metrics in these categories.

Although the KNN model demonstrated slightly higher overall accuracy, the Random Forest model showed greater stability, particularly in underrepresented outcomes such as PSMA‐PET and conventional imaging. It was therefore selected for deployment.

Figure S1 illustrates an example of a single decision tree trained during the model development process. It serves as a representative visualization of how clinical variables (e.g., PSA, ISUP, age) are hierarchically applied in the classification logic of the ensemble model.

### 3.3 Precision‐Recall curves

**FIGURE 2:**
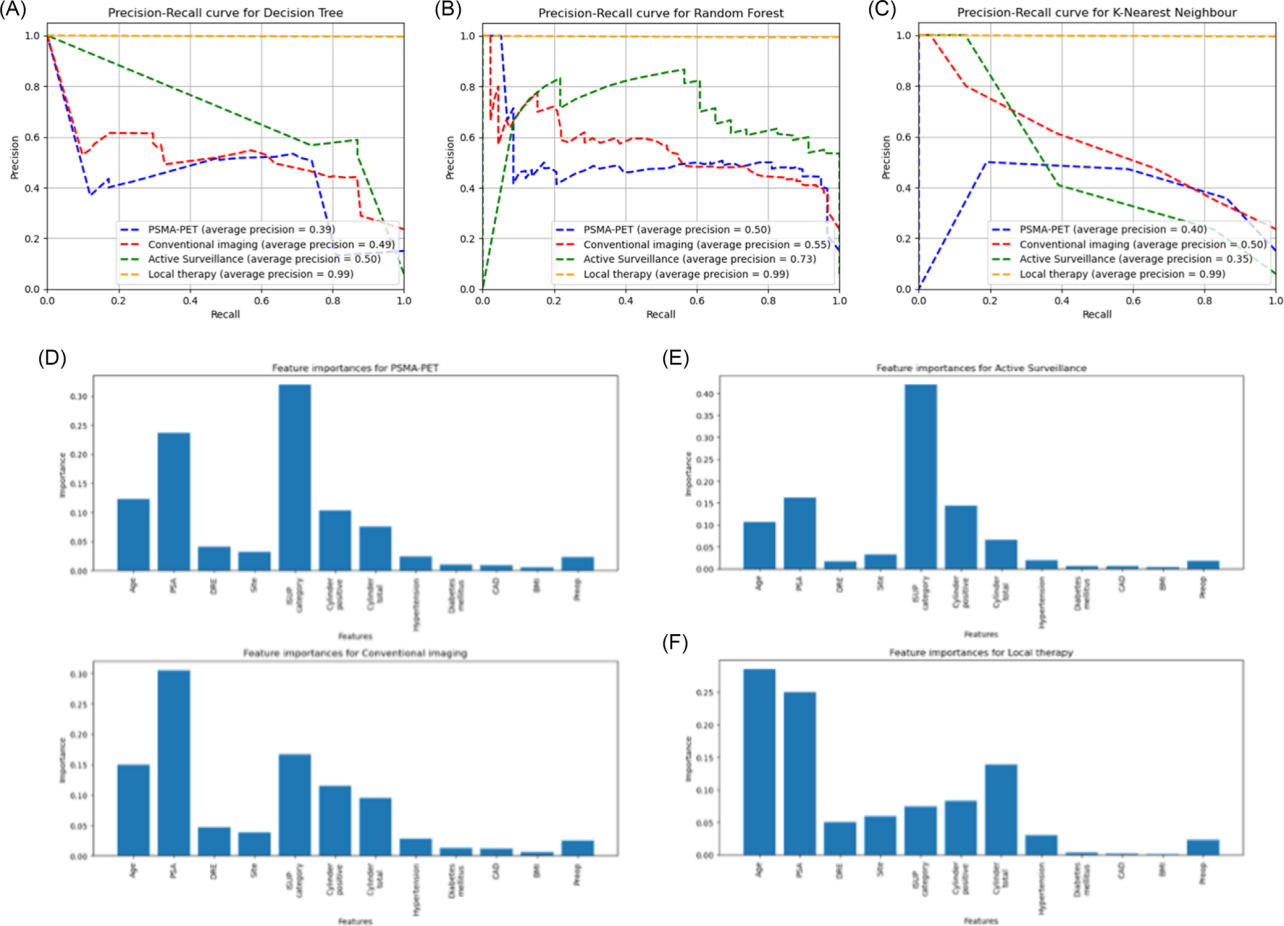
Performance metrics and feature relevance of machine learning models. (A–C) Precision‐recall curves for each classifier; (D–F) Feature importance visualizations for diagnostic and therapeutic outcomes based on Random Forest model.

The Precision‐Recall (PR) curves for the DT, RF and KNN models are illustrated in Figure 2A–C. The RF model maintained stable precision at higher recall values, while the DT and KNN models exhibited a steady decline in performance. This supports the selection of the RF model as the most reliable for tumour board recommendation prediction.

### 3.4 Model selection

Considering precision and recall metrics, the RF model was selected for further evaluation and deployment. The model demonstrated robust performance across different recommendation categories, ensuring consistency in predictions. The impact of guideline updates and the significance of feature selection were key considerations for future model refinements.

### 3.5 Feature importances

The feature importance analysis, calculated using Gini importance for the RF model, is presented in Table 3 and Figure 2D–F. Age, PSA levels and ISUP category were identified as the most influential predictors across all recommendation categories. While oncological factors significantly influenced model predictions, comorbidities had a comparatively minor impact.

**TABLE 3:** Feature importance of input variables in the Random Forest model: Relative contribution of clinical variables to model predictions across outcome categories, based on Gini importance. Oncological parameters such as PSA, ISUP grade and age had the highest influence, while comorbidities played a minor role.

Category	Age	PSA	DRE	Site of positive biopsy	ISUP category	Positive cylinder	Total cylinder	Hypertension	Diabetes mellitus	Cardiovaskular disease	Adipositas	Prior surgery
PSMA‐PET	0.12	0.24	0.04	0.03	0.32	0.1	0.08	0.02	0.01	0.01	0.005	0.02
Conventional imaging	0.15	0.31	0.05	0.04	0.17	0.12	0.09	0.03	0.01	0.01	0.006	0.02
Active surveillance	0.11	0.16	0.02	0.03	0.42	0.14	0.07	0.02	0.01	0.01	0.003	0.02
Local therapy	0.29	0.25	0.05	0.06	0.07	0.08	0.14	0.03	0.003	0.001	0.0004	0.02

## 4 DISCUSSION

We developed and validated a ML‐algorithm for automatically generating MTB recommendations for prostate cancer patients. The model was trained on a large dataset of historical tumour board decisions and was designed as a multi‐label classification task. We evaluated three different ML models—DT, RF and K‐Nearest Neighbour (KNN)—to determine the most effective approach. The RF model demonstrated the highest stability across multiple recommendation categories and was therefore selected for further development, including deployment in a simple front‐end web application.

### 4.1 Model performance and selection

The Random Forest model achieved the highest overall classification accuracy (66.3%), outperforming DT and KNN, particularly for underrepresented outcomes such as active surveillance and PSMA‐PET imaging. However, the overall accuracy remains limited and must be interpreted with caution. The modest performance highlights the inherent challenges of representing complex, nuanced clinical decisions as structured input variables. Moreover, expert recommendations vary over time and between physicians, particularly in the context of evolving clinical guidelines.

A notable limitation is the outcome category “local therapy,” which comprised radical prostatectomy or radiotherapy. This was predicted with near‐perfect precision and recall (F1‐score: 0.99) across all models, which reflects that a large proportion of patients were eligible for both treatment options. While this consistency aligns with guideline‐conform recommendations, it reduces the discriminative value of this prediction in a modelling context and may lead to overestimation of overall model performance.

The RF model maintained stable precision across higher recall thresholds, as shown by the precision‐recall curves, supporting its selection for further exploration. Nevertheless, we emphasize that the current accuracy level is not sufficient for clinical deployment and that further improvements in data quality and model structure are required.

### 4.2 Feature importance

Analysis of feature importance revealed that age, PSA and ISUP grade were the most influential variables across all recommendation categories. This aligns with clinical practice and confirms that the model captures key oncological factors in prostate cancer management. For diagnostic recommendations, PSA levels played a central role in PSMA‐PET selection, while ISUP grading and age were dominant in predicting conventional imaging. Active surveillance was most strongly associated with low ISUP grade, as expected. In contrast, comorbidities had limited influence on predictions, suggesting that their impact in real‐world decision‐making is not sufficiently captured in our structured dataset.

These findings demonstrate that the model reflects guideline‐based patterns but remains limited in its ability to account for more subtle or individualized clinical judgements.

### 4.3 Clinical relevance and potential impact

The application of AI‐based decision‐support tools in oncology has the potential to enhance efficiency, standardization and scalability of tumour board workflows. Our study illustrates that ML can replicate MTB decisions to a certain extent, especially for high‐frequency, guideline‐driven recommendations. The web‐based prototype demonstrates the feasibility of deploying such a system with minimal computational resources, which could make AI‐assisted recommendations more broadly accessible in the future.

Nonetheless, the current model should be understood as an initial proof‐of‐concept. It is not intended to replace clinical judgement, nor is it ready for clinical use. The accuracy achieved—while encouraging in parts—is insufficient for direct application in patient care. Clinicians must continue to validate all recommendations in the appropriate clinical context, particularly in borderline or complex cases where individual factors and shared decision‐making are critical.

### 4.4 Limitations and future directions

This study has several important limitations. First, the dataset was derived from a single academic institution, which limits generalizability. Tumour board practices are influenced by local protocols, physician preferences and regional adaptations of clinical guidelines. Future studies should incorporate multi‐centre data to improve external validity.

Second, the model relies exclusively on structured data extracted from standardized documentation. While this approach allows for efficient feature selection, it does not reflect the richness of narrative clinical information. Advanced natural language processing (NLP) methods, such as transformer‐based models, could potentially enhance the interpretability and precision of predictions by incorporating unstructured textual data.

Third, clinical guidelines and treatment strategies evolve over time. This dynamic environment necessitates regular model updates and retraining to remain in line with current standards. Prospective studies, ideally embedded in real‐world clinical workflows, will be essential to evaluate the practical utility and acceptance of ML‐based recommendation systems in oncology.

## 5 CONCLUSION

In summary, we developed and validated a machine learning model for the automated generation of multidisciplinary tumour board (MTB) recommendations in prostate cancer. Among the tested algorithms, the Random Forest model demonstrated the most robust performance and consistency across outcome categories. Feature importance analysis confirmed that classical oncological parameters—such as age, PSA level and ISUP grade—were the key drivers of prediction, reflecting established clinical decision‐making patterns.

The integration of this model into a web‐based application illustrates the technical feasibility of providing scalable, AI‐supported tools in oncological care. However, while the results are encouraging, the current model must be regarded as an early‐stage prototype. Its predictive performance—although reasonable—is not yet sufficient for clinical implementation. Further development is needed to enhance accuracy, address data imbalance and ensure adaptability to evolving guidelines.

Prospective validation in diverse clinical settings, combined with continuous model refinement and expansion of the underlying dataset, will be essential steps toward realizing the full potential of ML‐assisted decision support in prostate cancer care.

## AUTHOR CONTRIBUTIONS

Study conception: S.M., M.S., K.B. Data collection: H.G., S.M., A.R., F.L., F.F. Machine learning model development: M.S., S.M. Algorithm validation and testing: M.S., S.M. Clinical validation: K.B., R.H., F.L., F.F., M.S., S.M. Statistical analysis: S.M. Data preprocessing and feature selection: S.M., M.S., R.H., T.H. Manuscript writing: S.M., M.S. Manuscript review: All authors. Clinical supervision: C.T., M.S., T.H., M.M., S.M. Computational supervision: M.S., S.M.

## CONFLICT OF INTEREST STATEMENT

Matthias Miederer reports consulting fees from Novartis (Nürnberg, Germany), Roche (Grenzach‐Wyhlen, Germany), Telix (Melbourne, Australia) and Veraxa (Heidelberg, Germany). Christian Thomas reports consulting fees from Astellas, Janssen, Bayer and MSD; payment or honoraria for lectures, presentations, speakers bureaus from Astellas, Janssen, Bayer and MSD; support for attending meetings and/or travel from Janssen and Ipsen; and serves as a board member of the German Society of Urology. All other authors (Katharina Boehm, Fabian Funer, Roman Herout, Tobias Hölscher, Sherif Mehralivand, Marcus Sondermann) declare no conflicts of interest related to this work.

## Supporting information


**Table S1:** Clinical and Demographic Characteristics of All Tumour Board Cases (n = 6979; 2007–2024). Only a subset of these cases (n = 1929; from 2020 onwards) was used for model training and evaluation to ensure compatibility with contemporary guideline recommendations.
**Table S2:** Distribution of Tumour Board Recommendations in the Complete Dataset (n = 6979; 2007–2024) and the subset, titled as working cohort cases (n = 1929; from 2020 onwards). The subset was used for model training and evaluation to ensure compatibility with contemporary guideline recommendations.
**Figure S1:** Representative Structure of a Decision Tree Used in the Tumour Board Recommendation Model. Displayed is an individual decision tree trained as part of the ensemble model. It illustrates how patient‐specific input variables (e.g., PSA, ISUP grade) are used to derive classification outcomes.
