# Supplementary material for: A machine learning algorithm for automatic tumour board recommendations in prostate cancer patients
Source: BJUI Compass. 2025 Aug 18;6(8):e70066. doi: 10.1002/bco2.70066 (PMC12360893; doi:10.1002/bco2.70066)
Supplement: Supplementary file 1 — Table S1: Clinical and Demographic Characteristics of All Tumour Board Cases (n = 6979; 2007–2024). Only a subset of these cases (n = 1929; from 2020 onwards) was used for model training and evaluation to ensure compatibility with contemporary guideline recommendations. Table S2: Distribution of Tumour Board Recommendations in the Complete Dataset (n = 6979; 2007–2024) and the subset, titled as working cohort cases (n = 1929; from 2020 onwards). The subset was used for model training and evaluation to ensure compatibility with contemporary guideline recommendations. Figure S1: Representative Structure of a Decision Tree Used in the Tumour Board Recommendation Model. Displayed is an individual decision tree trained as part of the ensemble model. It illustrates how patient‐specific input variables (e.g., PSA, ISUP grade) are used to derive classification outcomes. [file BCO2-6-e70066-s001.docx]

Supplemental Material

| **Characteristic** | **N = 6,979^1^** |
| --- | --- |
| Date of Tumorboard | 2011-12-21, 2020-05-06 |
| Age at diagnosis | 66 (61, 70) |
| iPSA Value | 7 (5, 11) |
| **Digital rektal examination** | |
| cT1 | 5,572 (83%) |
| cT2 | 886 (13%) |
| cT3 | 240 (3.6%) |
| cT4 | 19 (0.3%) |
| **Gleason Grading** | |
| 6 | 1,943 (28%) |
| 7 | 3,488 (50%) |
| 7a | 2,466 (36%) |
| 7b | 1,021 (15%) |
| 8 | 901 (13%) |
| 9 | 510 (7.4%) |
| 10 | 61 (0.9%) |
| **ISUP-Grading** | |
| 1 | 1,943 (28%) |
| 2 | 2,466 (36%) |
| 3 | 1,021 (15%) |
| 4 | 901 (13%) |
| 5 | 571 (8.2%) |
| **Prostate Biopsy** | |
| Removed Cylinder | 12.0 (11.0, 15.0) |
| Positive Cylinder | 4.0 (2.0, 6.0) |
| Positive Site of Cylinder | |
| Both sides | 3,099 (48%) |
| left | 1,737 (27%) |
| right | 1,645 (25%) |
| **Site illnes** |  |
| Hypertension | 3,097 (44%) |
| Diabetes mellitus | 608 (8.7% |
| Coronary Heard Disease | 235 (3.4%) |
| Adipositas (BMI > 35) | 267 (3.8%) |
| ^1^ Median (Q1, Q3); n (%) | |
| **Supplementary Table 1:** Clinical and Demographic Characteristics of All Tumor Board Cases (n = 6,979; 2007–2024). Only a subset of these cases (n = 1,929; from 2020 onwards) was used for model training and evaluation to ensure compatibility with contemporary guideline recommendations. | |

|  | **Overall Kohort** | **Working Cohort** |  |
| --- | --- | --- | --- |
|  | **N = 6,9791** | **N = 1,929** |  |
| **Diagnostics**  CT-Scan | | 1,050 (15%) | 451 (23%) |
| MRI | | 213 (3.1%) | 90 (4.7%) |
| PSMA-Scan | | 356 (5.1%) | 291 (15%) |
| **Therapeutics** ADT | | 488 (7.0%) | 379 (20%) |
| Prostatectomy | | 6,937 (99%) | 1,909 (99%) |
| Nervesparing Surgery | | 423 (6.1%) | 0 (0%) |
| Pelvic Lymphnodedissection | | 1,482 (21%) | 390 (20%) |
| SBRT | | 6,900 (99%) | 1,900 (98%) |
| lymphvessels | | 75 (1.1%) | 30 (1.6%) |
| brachytherapy | | 445 (6.4%) | 12 (0.6%) |
| Active Surveillance | | 299 (4.3%) | 112 (5.8%) |
| 1 n (%) | | | |
| **Supplementary Table 2** Distribution of Tumor Board Recommendations in the Complete Dataset (n = 6,979; 2007–2024) and the subset, titled as working cohort cases (n = 1,929; from 2020 onwards). The subset was used for model training and evaluation to ensure compatibility with contemporary guideline recommendations. | | | |

|  |
| --- |
| 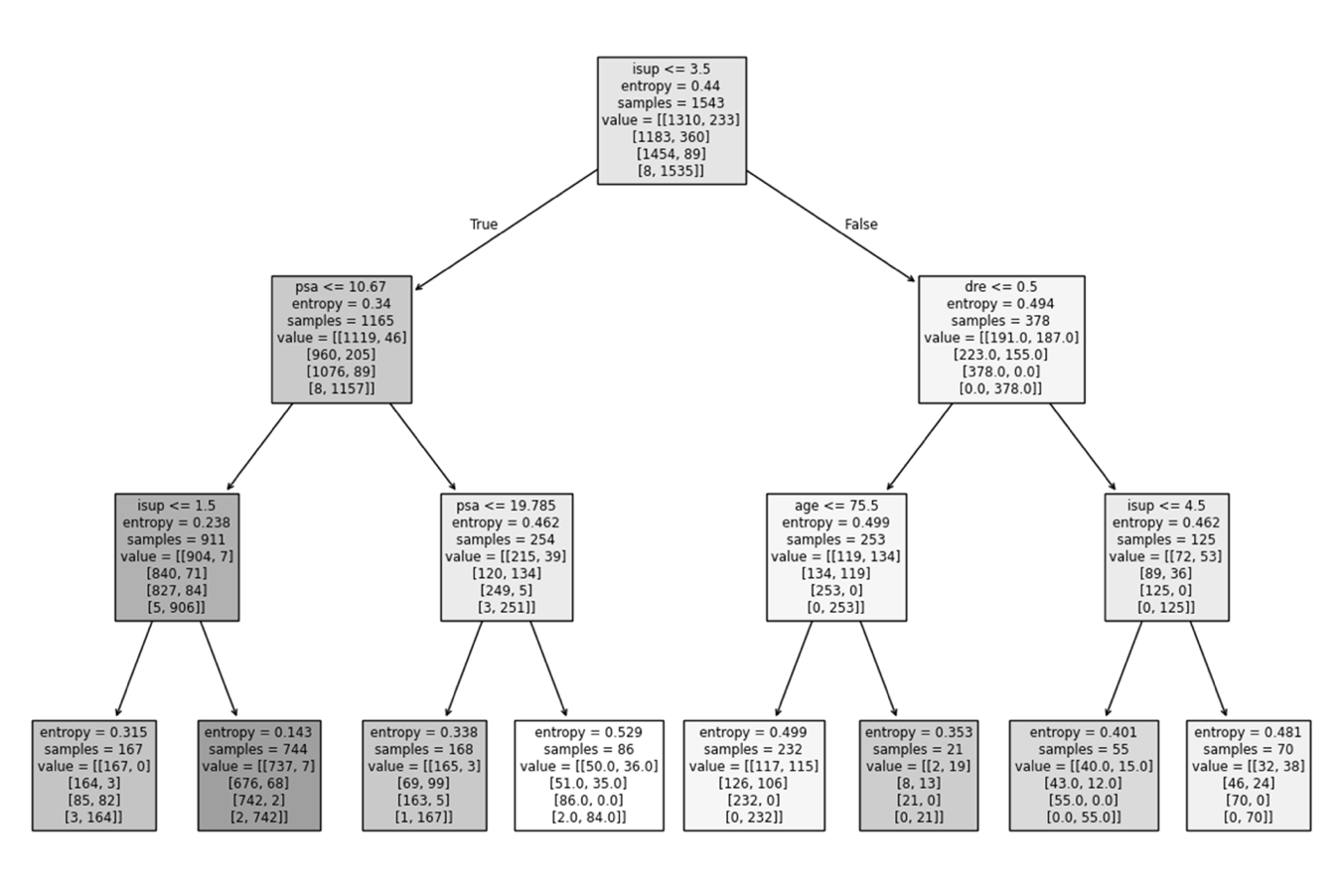 |
| **Supplementary Figure 1**: Representative Structure of a Decision Tree Used in the Tumor Board Recommendation Model. Displayed is an individual decision tree trained as part of the ensemble model. It illustrates how patient-specific input variables (e.g., PSA, ISUP grade) are used to derive classification outcomes. |
